# A Prism Method for Optical Glomerular Mapping of the Medial Olfactory Bulb in Mice

**DOI:** 10.3389/fncir.2019.00079

**Published:** 2019-12-20

**Authors:** Ryota Homma, Shin Nagayama

**Affiliations:** Department of Neurobiology & Anatomy, McGovern Medical School at the University of Texas Health Science Center at Houston, Houston, TX, United States

**Keywords:** sensory maps, glomerulus, olfactory sensory neuron, optical imaging, mirror symmetry, bulbectomy

## Abstract

The processing of odor input in the brain begins in the olfactory bulb (OB), where odor information is represented by combinations of active glomeruli. Each glomerulus is associated with a specific odorant receptor type, of which there are ~1,000 in mice; thus different odors activate different subsets of glomeruli. Most receptor types have duplicate lateral and medial glomeruli in each of the left and right OBs. The two sets of glomeruli form separate but mirror-symmetric glomerular maps. It is not known whether the odor representations in these paired maps are exact copies of each other or potentially encode additional information. Previous studies of glomerular odor representations were mostly limited to the lateral map because the medial map is inaccessible with high-resolution activity mapping techniques, such as optical imaging. To address this, we developed a method for optical imaging of the medial bulb by replacing the contralateral bulb with a right-angle prism that has a mirror coating on the hypotenuse. With this method, we performed calcium imaging of corresponding subsets of glomeruli in the lateral map at the dorsal surface and the medial map at the medial wall. Thus, we demonstrate an experimental model system for comparing odor representations in these redundant sensory maps, enabling a better understanding of the role of paired maps and the neuronal coding of odor stimuli.

## Introduction

In mammals, glomeruli in the olfactory bulbs (OBs) receive input from olfactory sensory neurons (OSNs). Each OSN expresses only one type of odorant receptor, and each glomerulus receives input from the OSNs of a single receptor type (Mori and Sakano, 2011; Murthy, 2011). Thus, each glomerulus is associated with a specific type of odorant receptor, and odor information in the OB is represented by the activity of combinations of glomeruli. In rodents, axons from the most receptor types of OSNs converge to two glomeruli in each bulb: one glomerulus on the lateral side and one on the medial side. The two sets of glomerulus populations are arranged to form two mirror-symmetric maps (Mori and Sakano, 2011; Murthy, 2011), one in the dorsal to lateral aspects and another in the medial to ventral aspects of the OBs (hereafter referred to as lateral and medial maps, respectively). The dorsal boundary between maps is approximately from the posterior end of the dorsal OB to the anterior-ventral end of the medial OB, although it has yet to be precisely determined.

Although the mirror-symmetric maps have been demonstrated with several different approaches (Nagao et al., 2000; Johnson and Leon, 2007; Zapiec and Mombaerts, 2015), their roles in odor coding remain unclear. For example, the two maps may represent highly redundant odor representations for robust signals or diversified odor representations for greater capacity and/or precision of coding. To address this, the glomerular odor representations in the two maps must be compared. However, there are no adequate techniques to map the glomerular activity in the medial map. Two methods that enable activity mapping of the entire OB, namely, functional magnetic resonance imaging (Xu et al., 2003) and 2-deoxyglucose mapping (Johnson and Leon, 2007), lack the spatial resolution to distinguish single glomeruli. Optical imaging techniques are limited to the dorsal OB or the lateral OB (Igarashi and Mori, 2005). The medial portion can only be imaged by the removal of the obstructing contralateral OB (Shirasu et al., 2014).

In the present study, we developed a method to optically access the medial OB following bulbectomy (Shirasu et al., 2014), *via* a right-angle prism with a mirror coating on the hypotenuse. This type of prism has been used to obtain lateral views of microscopy specimens, including inside of brain tissue (Murayama et al., 2007; Chia and Levene, 2009; Andermann et al., 2013) as well as lateral aspects of brain surface (Heys et al., 2014; Low et al., 2014). We developed a protocol to replace the contralateral OB with a prism and performed optical imaging in the medial OB. Using this method in the urethane-anesthetized mice, we recorded the odor-evoked calcium responses of glomeruli in the posterior-medial OB (medial map) and found the corresponding glomerular ensembles in the anterior-dorsal OB (lateral map). This technique provides an opportunity to optically monitor and/or manipulate glomerular ensembles within mirror-symmetric maps for comparison.

## Materials and Methods

### Mouse Lines

Two mutant mouse lines were used in this study. The first line (mOR-EG/Thy1-GCaMP6f mice) was obtained by crossing mOR-EG transgenic mice (Oka et al., 2006: gifted from Dr. Kazushige Touhara) and Thy1-GCaMP6f mice (GP5.11, JAX #24399; Dana et al., 2014). The second line (OMP-GCaMP6f mice) was obtained by crossing OMP-Cre mice (JAX #6668; Li et al., 2004) and Cre-dependent GCaMP6f reporter mice [Ai95D (C57BL/6J), JAX #28865; Madisen et al., 2015]. Five mOR-EG/Thy1-GCaMP6f mice (10–19 weeks old; one female) and five OMP-GCaMP6f mice (6–14 weeks old; two females) were used in this study.

### Surgical Procedure

All animal procedures were conducted in accordance with an animal protocol approved by the Institutional Animal Care and Use Committee of the University of Texas Health Science Center at Houston.

Animals were anesthetized with an intraperitoneal injection of urethane [6% (w/v), 20 μl/g body weight for the initial injection]. The depth of anesthesia was monitored *via* toe pinch, and additional injections (1/6–1/3 dose of the initial injection) were administered to maintain the appropriate level of anesthesia. Animals breathed freely throughout the entire experiment. Rectal body temperature was maintained at between 36.0 and 37.0°C with a heating pad.

For each mouse, an incision in the scalp was made to expose the skull. Connective tissue attached to the skull was carefully removed, and a metal head plate with an elliptic opening [7 mm in the anterior-posterior axis and 5 mm in the medial-lateral axis; CP-1 (Narishige), with slight customization] was attached with cyanoacrylate glue and dental acrylic. A dental drill was used to thin the skull over each of the OBs as well as over the midline blood vessel to transparency when moist. For the bulbectomy, the blood vessel between one OB and the frontal cortex was blocked at the medial and lateral ends by pushing bone wax through a small hole made with the dental drill. The skull above the blocked blood vessel was removed to provide enough space for the prism. The skull over the contralateral OB was further thinned around the edges and removed. The dura mater covering the dorsal bulb was cut at the medial end of the OB and removed. Bulbar tissue was then carefully removed by aspiration. Once all bleeding had stopped (influx of cerebrospinal fluid was tolerated), the dura mater covering the medial wall of the bulbectomized OB was either removed or peeled down ventrally; the dura mater covering the remaining OB was kept intact. The bulbectomized space, as well as the dorsal surface of the remaining OB, was filled with agarose (1.0%). A portion of agarose was removed from the posterior-medial end of the bulbectomized space, and a 1.5 mm coated micro prism (4531-0023; Tower Optical) was placed. The remaining OB and the prism were covered with a precut coverslip (approximately 3.0 mm in the anterior-posterior axis, 3.5 mm in the medial-lateral axis) fixed in place with cyanoacrylate glue and dental acrylic.

After the surgical procedure was fully established, the success rate of the experiment was 50% (8/16). Out of eight unsuccessful experiments, three were failures in bulbectomy (e.g., the tissue of the recording side was damaged). In the remaining five cases, the general condition of the anesthetized animal became unsatisfactory before or during the recording, although the surgical procedure was successfully completed.

### Optical Imaging

Optical imaging was conducted with a fluorescence microscope (Olympus) equipped with a high-speed charge-coupled-device camera (NeuroCCD-SM256; RedShirtImaging). The microscope was set up on a vibration-isolating air table (Newport). All recordings were conducted under urethane anesthesia immediately after the surgical procedure described in the previous subsection. Animals breathed freely and rectal body temperature was kept maintained with a heating pad. The animal was secured with an angle-adjustable animal holder (MAG-3; Narishige) attached to the affixed head plate. A white-color light-emitting diode module (MCWHL2-C1; Thorlabs) and a standard green fluorescent protein (GFP) filter cube (GFP-4050A-OMF-ZERO, 466/495/525 nm exciter/DM/emitter; Semrock) were used for imaging with a 10×/0.3 NA objective lens (Olympus) and a 0.35× tube lens (Olympus), resulting in a field of view of 1.75 × 1.75 mm^2^ (128 × 128 pixels). Each imaging trial comprised a 13 s recording at 125 Hz (1,625 frames). Trials were separated by at least 60 s. In every mouse, recording from the medial OB *via* the prism preceded recording from the dorsal OB. The animals’ breathing cycles were recorded with a piezo-element placed under the chest.

### Odor Presentation

Odorants were presented with a custom-built device described previously (Kikuta et al., 2013; Homma et al., 2019). Briefly, the presentation of a continuous flow of a mixture of odorous gas (diluted odorant bubbled with 100% nitrogen, 100 ml/min) and carrier gas (filtered air, 400 ml/min) was regulated *via* a vacuum near the tip of the applicator. Brief discontinuation of the vacuum *via* an electric signal resulted in the presentation of the odorous gas for 2 s. The onset of the command signal was synchronized to the end of the animal’s inhalation to prevent odorant delivery in the middle of inhalation. The baseline prestimulus period was at least 4 s.

Odorants were presented in a fixed order for four blocks. When multiple concentrations of the same odorant were presented (e.g., 0.02%, 0.2%, and 2%), lower concentrations were presented first. The concentrations of odorants were adjusted *via* liquid dilution with mineral oil and 1:5 flow dilution to elicit strong but not saturating responses. Thus, nominal concentrations of odorants are indicated as 1/5 of the liquid dilution (e.g., 0.2% when an odorant was liquid-diluted to 1%). The odorants and concentrations used were as follows: aliphatic aldehydes with carbon chain lengths of 3–7 (3–7CHO; 0.2%), benzaldehyde (2%), salicylaldehyde (2%), ethyl tiglate (2%), propyl butyrate (PB; 0.2%), ethyl caproate (EC; 2%), methyl valerate (MV; 0.2%), butyric acid (10%), isovaleric acid (10%), and eugenol (20%). All odorants and mineral oil were purchased from Sigma-Aldrich.

### Data Analysis

As photobleaching was observed in some instances, all data were preprocessed with a custom-written macro (script) for ImageJ/Fiji (Schindelin et al., 2012) for photobleaching correction. The macro divided the images of 128 × 128 pixels into 1,024 blocks of 4 × 4 pixels, and in each block, the first 480 frames (3.8 s) and the last five frames (0.04 s) were fitted to a single exponential curve. This block processing was chosen to manage processing time, spatial heterogeneity of photobleaching, and the signal-to-noise ratio of data used for curve fitting. The inclusion of the last few frames in curve fitting minimizes obvious overcorrections due to overfitting to the early frames, but the contribution from the last frames is not sufficient to alter the fitted curve at the tail when the curve is distant from the baseline. For each pixel in the block, the exponential curve was multiplied by a constant so that the initial value matched the pixel intensity of the first frame, and then the scaled curve was subtracted from the entire time course of the pixel. In a few cases (<1%), this resulted in block noise; such trials were excluded from further analyses. The activity maps (described below) were not qualitatively different from those without the photobleaching corrections.

All activity map data represent an average from 3 to 4 trials. First, breathing cycle data were analyzed to determine the onset of the first inhalation cycle after the stimulus presentation. All imaging data were aligned to this time point, and data from the time-aligned raw image series of four trials were averaged. The trial-averaged stacks were used to calculate a response image, which was an average of 200 frames (1.6 s) imaged at the onset of the first inhalation cycle with stimulus, and a baseline image, which was an average of 125 frames (1.0 s) imaged 1.4–0.4 s before the onset of the first inhalation. Activity maps were obtained by subtracting the baseline image from the response image and then dividing by the baseline image (i.e., ΔF/F_0_ calculation). No statistical analyses were performed in this study.

## Results

### Optical Imaging of Medial OB Through a Prism

**Figure 1:**
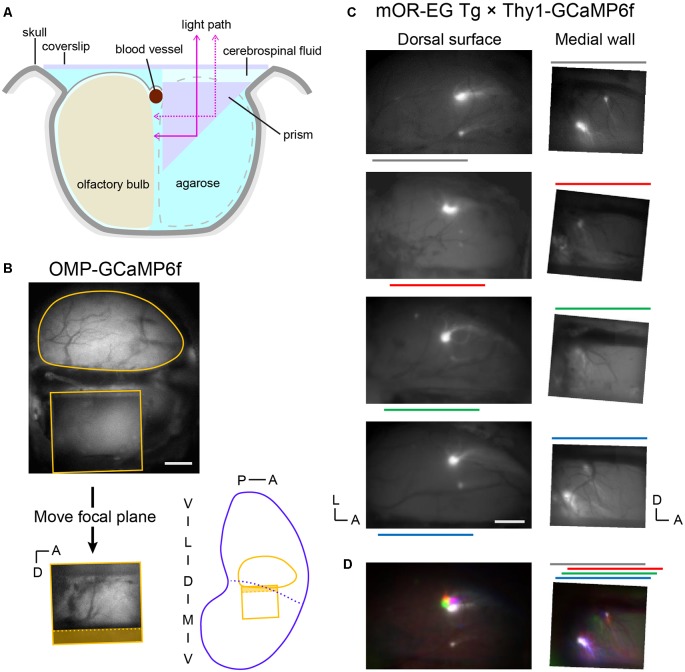
Optical imaging of the posterior-medial olfactory bulb (OB) through a prism. **(A)** Schematic illustration of the OB preparation (coronal view). One OB was replaced by a 1.5 mm prism with a mirror coating on the hypotenuse and agarose. The dorsal surface of the remaining OB (recording side) and the prism were covered with a coverslip. **(B**, top**)** Top view of a preparation visualizing the fluorescence from an OMP-GCaMP6f mouse. The top surface of the prism (rectangle) is visible below the dorsal surface of the left OB (round contour) in the image. The OB surface is not visible through the prism because it is out of focus. (Bottom left) Image of the medial OB through the prism. The focal plane was approximately 1 mm lower than that of the dorsal surface. Note that the dorsal-ventral axis is inverted in this mirror image. The shaded area corresponds to the side view of the midline blood vessel (dotted line) and empty space above it. (Bottom right) Approximate positions of the dorsal and medial fields of view in an unrolled OB map. Blue dotted line indicates the probable boundary of the medial and lateral OB maps. **(C**, left**)** Dorsal surfaces of OBs from four of five mOR-EG transgenic mice in which the glomeruli are labeled with green fluorescent protein (GFP; Oka et al., 2006). The left and bottom edges of each image are aligned to the posterior and medial ends of the OB, respectively. Color lines indicate the anterior-posterior position of the prism on the contralateral side. Surface blood vessel patterns were visualized by weaker fluorescence from the GCaMP6f expressed in glutamatergic neurons. (Right) The medial surface of the same OBs. These images were vertically flipped to present the noninverted dorsal-ventral axis. **(D**, left**)** The four images in panel **(C)** were colored and superimposed to show their relative positions. Colors are matched to the bars in panel **(C)**. (Right) Corresponding superimposed image for the medial OB. The relative positions of individual images were adjusted along the anterior-posterior axis, as indicated in the color bars above the image, to compensate for the differences in the anterior-posterior positioning of the prism (relative to the posterior end of the OB). A, anterior; D, dorsal; L, lateral; M, medial; P, posterior; V, ventral. Scale bars: 0.5 mm.

To gain optical access to the medial OB, we replaced one OB with a right-angle prism that worked as a mirror (Figure 1A). This enabled us to clearly observe GCaMP6f or GFP expression in the medial OB under a fluorescence microscope (Figures 1B,C). The focal plane for the medial OB through the prism was approximately 1 mm below the focal plane for the dorsal OB in our preparations. Thus, the top of the prism was approximately 0.5 mm above the dorsal surface because the light-path length in the prism was 1.5 mm. All experiments were conducted in an acute procedure so that the recording started immediately after the surgical procedure. We successfully recorded up to ~150 trials that took for up to 7 h.

### Posterior-Medial OB Imaged Through a Prism Corresponds to the Anterior-Dorsal OB in the Mirror-Symmetry Maps

On the basis of what has been described in the literature, we anticipated that maps in the posterior-medial OB correspond to maps in the anterior-dorsal OB. Thus, we crossed mOR-EG mice, in which a pair of GFP-labeled glomeruli appear in the anterior-dorsal OB and posterior-medial OB (Oka et al., 2006), with Thy1-GCaMP6f mice so that the entire OB was fluorescent (Figure 1C). Although the fluorescence spectra of GFP and GCaMP6f overlap, the GFP signals were much brighter than the GCaMP6f signals. The positions of GFP-labeled glomeruli were stable among animals, as determined by aligning the images of the dorsal OBs from different animals to the posterior and medial ends of the OBs. In the medial OB, these glomeruli were sometimes observed in the ventral-posterior end of the prism window. We frequently observed a smaller subglomerulus dorsal and anterior to the GFP-expressing main glomerulus. In cases where we did not clearly see a glomerulus in the ventral-posterior end, we found that the prism had been placed in a relatively anterior position, and the putative subglomerulus appeared relatively posterior in the window (Figure 1C). We speculate that in these cases, the main glomerulus was posterior to the edge of the prism window. After correcting for the differences in the relative positions of the prism, both the main glomeruli and subglomeruli appeared in respective overlapping subregions (Figure 1D). These observations strongly suggest that the medial OB within the prism window is matched with the anterior-dorsal OB in the mirror-symmetry maps.

### Glomerulus-Level Mirror Symmetry Between the Anterior-Dorsal and Posterior-Medial OB

**Figure 2:**
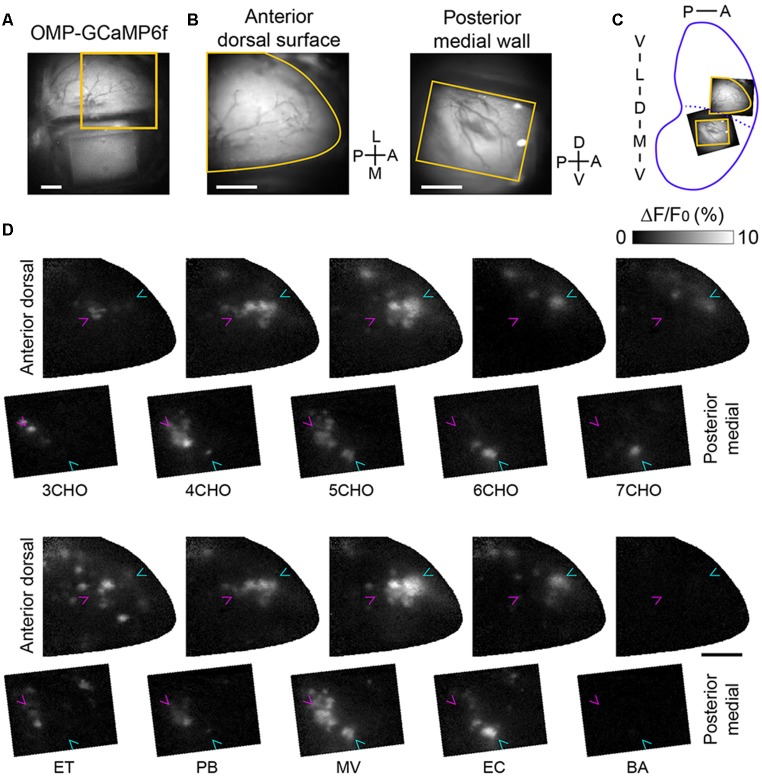
Calcium imaging of corresponding glomerular ensembles in the anterior-dorsal and posterior-medial OB. **(A)** An image of the dorsal surface as well as the prism placed on the contralateral side. The orange square indicates the field of view for the calcium imaging of the anterior-dorsal OB. **(B)** Fields of view for calcium imaging in the anterior-dorsal OB (left) and posterior-medial OB (right). The image of the posterior-medial OB was vertically flipped for illustration. Orange contours indicate the edge of cranial opening or prism window presented in panel **(D)**. **(C)** Approximate positions of the dorsal and medial fields of view are indicated in an unrolled OB map. **(D)** Activity maps of odor-evoked calcium responses in the fields of view shown in panel **(B)**. The first and third rows are activity maps of the anterior-dorsal OB, and the second and fourth rows are respective activity maps of the posterior-medial wall. The relative position of anterior-dorsal and posterior-medial activity maps are the same as orange contours in panel **(C)**. Magenta and cyan arrowheads indicate the positions of glomeruli most sensitive to 3CHO and 6CHO, respectively, in each field to facilitate comparison. Representative data from three experiments are presented. A, anterior; D, dorsal; L, lateral; M, medial; P, posterior; V, ventral; 3–7CHO, aliphatic aldehydes with carbon chain lengths of 3–7, respectively; ET, ethyl tiglate; PB, propyl butyrate; MV, methyl valerate; EC, ethyl caproate; BA, benzaldehyde. Scale bars: 0.5 mm.

If the positions of the GFP-labeled mOR-EG glomeruli correspond precisely to those in the mirror-symmetry maps, the sites next to the dorsal GFP glomerulus and the medial GFP glomerulus should also correspond and thus represent a set of homologous glomeruli. To determine whether the odorants that activate the anterior-dorsal OB also activate the posterior-medial OB, we performed experiments in which sets of odorants, including ones known to activate the anterior-dorsal OB (Mori et al., 2006; Matsumoto et al., 2010), were presented (Figure 2). In the posterior-medial OB, a subset of the tested odorants activated glomeruli in the posterior half of the window. We also noticed that a similar number of glomeruli in the center of the anterior-dorsal OB were activated by the same odorants. We found that a few pairs of odorants activated nearly identical sets of glomeruli in the anterior-dorsal and posterior-medial OB (compare 4CHO vs. PB, 5CHO vs. MV, and 6CHO vs. EC in Figure 2C), further suggesting that these are homologous sets of glomeruli. Among the odorants tested, eugenol elicited much weaker but unambiguous response in the anterior dorsal OB in two of three mice tested but did not elicit a detectable response in the posterior medial OB. Benzaldehyde, salicylaldehyde, butyric acid, and isovaleric acid did not induce clear responses in the posterior-medial OB or in the putatively corresponding subregion of the dorsal-anterior OB, although these odorants activated glomeruli in the other part of the dorsal OB (data not shown).

There are cases in which the position of active glomeruli gradually shifts according to the carbon chain length of a specific class of odorant molecules, referred to as a local chemotopic glomerular representation (Uchida et al., 2000), though how this may be related to odor coding is unclear (Murthy, 2011). Thus, we carefully compared glomerular activity maps and observed chemotopic representation with aliphatic aldehydes (3CHO to 7CHO), both in the anterior-dorsal and posterior-medial OB (Figure 2C, note the magenta and cyan arrowheads pointing to the glomeruli most sensitive to 3CHO and 6CHO, respectively). These findings suggest that the precision of the mirror symmetry is similar to that found between the left and right dorsal OBs (Belluscio and Katz, 2001; Soucy et al., 2009).

### Detection Thresholds for the Glomerular GCaMP Signals Are Comparable Between the Anterior-Dorsal and Posterior-Medial OB

**Figure 3:**
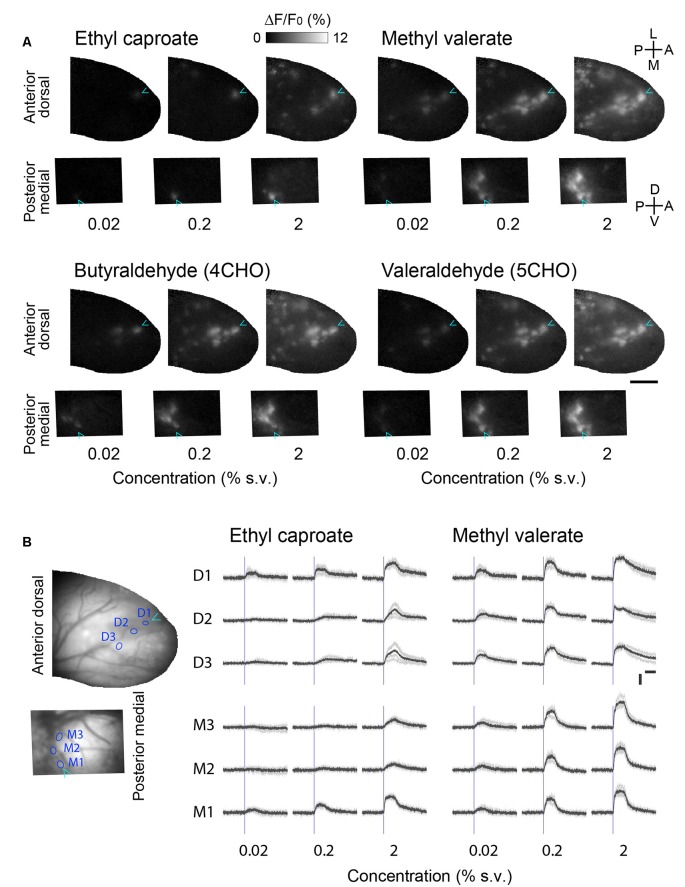
Concentration-response relationships in the corresponding glomerular ensembles in the anterior-dorsal and posterior-medial OB. **(A)** Activity maps in the anterior-dorsal and posterior-medial OBs of OMP-GCaMP6f mice in response to three different concentrations of four monomolecular odorants. The activity maps of the posterior-medial OB are vertically flipped for illustration. Relative positions of dorsal and medial fields of view are arranged in the same way as in Figure 2D. Cyan arrowheads indicate the position of the glomerulus most sensitive to ethyl caproate. Representative data from two experiments are presented. Concentration is presented as a percentage of saturated vapor (for detail see “Materials and Methods” section). A, anterior; D, dorsal; L, lateral; M, medial; P, posterior; V, ventral. Scale bar: 0.5 mm. **(B)** Example time courses of glomerular calcium signals presented in **(A)**. Three glomeruli in the anterior dorsal bulb (D1–3) and three in the posterior medial bulb (M1–3) are shown for two odorants. The positions of glomeruli are indicated as circles in the left images. Gray traces represent each of four individual trials and black traces represent the average of those four trials. Blue vertical lines indicate the onset of first inhalation after the stimulus presentation (2 s). Vertical scale bar: 5% ΔF/F; horizontal scale bar: 2 s.

The OSNs innervating the anterior-dorsal OB and the posterior-medial OB are distinct populations that are spatially segregated in the olfactory epithelium (Schoenfeld and Cleland, 2005, 2006). To determine whether the differing positions in the epithelium affect the responsiveness of glomeruli in the anterior-dorsal and posterior-medial OB, we recorded glomerular responses to various concentrations of the odorants (Figure 3). We found that the number of glomeruli in the subregions (the center of the anterior-dorsal OB and the posterior half of the prism window for the medial-posterior OB) that responded were always consistent across concentrations. Although we were unable to identify the exact pairs of homologous glomeruli, it is unlikely that the glomeruli in the anterior-dorsal and posterior-medial OB represent different sets (i.e., are nonhomologous) or that these glomeruli differ in responsiveness.

## Discussion

In this study, we established a method to optically access the posterior-medial OB through a prism. Using this technique, we identified pairs of corresponding glomerular ensembles in the lateral and medial maps that can be used to study the function of mirror-symmetric glomerular maps in the rodent OB as an experimental model system.

This is not the first report of optical imaging of the medial OB (Shirasu et al., 2014). However, our method enables both the dorsal and medial OB to be imaged in quick succession. With a low-magnification configuration, dorsal and medial optical fields can be switched just by moving the z-axis. In our preparations, the focal planes are approximately 1 mm apart, mainly due to the extra light path of the prism. This distance does not impede the switching of focal planes but may preclude simultaneous recording of the dorsal and medial OB. The high compatibility of our technique with ordinary microscopes enables the use of a wider variety of optical tools, such as patterned optogenetic stimulations (Dhawale et al., 2010) or two-photon microscopy. In particular, previous studies demonstrated two-photon imaging of individual neurons through an implanted prism in various brain areas of behaving animals (Andermann et al., 2013; Heys et al., 2014; Low et al., 2014). Thus recording from individual principal neurons in the OB (mitral/tufted cells) in behaving animals is potentially feasible, although our current protocol is designed for acute experiments and we have not tested long-term clarity of cranial window. We speculate that some refinements of the protocols are needed for the long-term stability and clarity of the cranial window, with careful design consideration to overcome the limited space around the OB.

Currently, the field of view through the prism is limited to the dorsal part of the medial OB. We chose a 1.5 mm prism (1.5 × 1.5 × 1.5 mm) because this was the largest that would fit into the bulbectomized space in an adult mouse. As the edge of the cranial opening limited the depth of the prism in our configuration, a larger prism would require a more extensive craniotomy, which would be more invasive. Alternatively, a smaller prism or two small prisms arranged side by side, could be used to image the anterior part of the medial OB, including the boundary between medial and lateral maps. Smaller prisms may also be useful for imaging more ventral parts of the medial OB. Another caveat regarding this method is that it requires ablation of one OB, which eliminates inter-bulbar connections (Schoenfeld and Macrides, 1984; Yan et al., 2008; Grobman et al., 2018). In the future, the impact of bulbectomy needs to be assessed, for example, by comparing glomerular odor representations in the dorsal OB before and after the contralateral bulb is removed.

In summary, we identified the posterior-medial OB and anterior-dorsal OB as corresponding subregions that share the same glomerular ensembles. Odor representations in the anterior-dorsal OB have been extensively studied (Rubin and Katz, 1999; Uchida et al., 2000; Wachowiak and Cohen, 2001; Spors and Grinvald, 2002). These studies provide information that will become a firm basis for future studies with our method to compare odor representations between the lateral and medial maps in this model system.

## Data Availability Statement

The datasets generated for this study are available on request to the corresponding author.

## Ethics Statement

The animal study was reviewed and approved by Institutional Animal Care and Use Committee of the University of Texas Health Science Center at Houston.

## Author Contributions

RH and SN designed the experiments and wrote the manuscript. RH performed the experiments and analyzed the data.

## Conflict of Interest

The authors declare that the research was conducted in the absence of any commercial or financial relationships that could be construed as a potential conflict of interest.
